# Do Moral Emotions Interact with Self-Control and Unstructured Socializing in Explaining Rule-Breaking Behavior Committed Together with Friends?

**DOI:** 10.3390/children11070766

**Published:** 2024-06-25

**Authors:** Sara-Marie Schön, Monika Daseking

**Affiliations:** Developmental and Educational Psychology/Humanities and Social Sciences, Helmut-Schmidt-University (University of the Federal Armed Forces Hamburg), 22043 Hamburg, Germany

**Keywords:** moral emotions, situational action theory, rule-breaking behavior, self-control, unstructured socializing

## Abstract

Previous research has shown that moral emotions interact with self-control and unstructured socializing in explaining rule-breaking behavior. High levels of moral emotions appear to weaken the effects of both self-control and unstructured socializing, in explaining rule-breaking behavior. The current study examined whether these interactions also affect rule-breaking behavior that is explicitly committed with friends. In addition, three operationalizations of moral emotions were distinguished. Data were collected from *N* = 169 adolescents (54% female; mean = 14.95 years; SD = 1.7) using a self-report questionnaire battery. Results indicate that high levels of anticipated emotions in moral conflicts (AEMC) attenuate the effect of low self-control on one’s own rule-breaking behavior. In contrast, high levels of both guilt- and shame-proneness enhanced the effect of unstructured socializing on one’s own and rule-breaking with friends. The limitations of the study, ideas for future research, and practical implications are also discussed.

## 1. Introduction

A variety of theoretical frameworks may be addressed to elucidate the phenomenon of rule-breaking behavior. These include psychopathological, psychological, and criminological approaches. The significance of moral emotions in explaining rule-breaking behavior is not only apparent in psychological research, but also in criminology, as evidenced by Situational Action Theory [1,2]. Within SAT, crime is analyzed as moral action, i.e., ‘actions (including intentional inactions) guided by valued-based and emotionally grounded rules of conduct about what is the right or wrong thing to do in particular circumstances’ [2] (p. 262). This is a key strength of the SAT—its assumptions can be applied to all forms of rule-breaking behavior. On this basis, the term ‘rule-breaking behavior’ is used to refer to dissocial, amoral, and delinquent behavior. All three behavioral outcomes involve breaking rules. Dissocial behavior involves breaking a social rule, such as not keeping a promise or threatening someone. Amoral behavior involves breaking a moral rule, such as lying. Finally, delinquent behavior involves breaking a rule relevant to the law, such as stealing.

SAT assumes that rules are only broken if breaking the rules is perceived as an alternative course of action, which is largely dependent on the individual’s morality [1,2]. Consistent with this research has shown that moral emotions attenuate the effects of risk factors for rule-breaking behavior. For example, low self-control and rule-breaking friends were only related to rule-breaking behavior among adolescents with low levels of moral emotions [3,4].

These findings serve as a starting point for the present study. It examines whether the ‘buffering effect’ of moral emotions also affects the rule-breaking behavior that is explicitly committed together with friends. In addition, the three operationalizations of moral emotions are distinguished: guilt- and shame-proneness as well as emotions that are anticipated in moral conflicts. The former is particularly relevant for the measurement of rule-breaking behavior in adolescence and the latter for its prevention. Rule-breaking behavior is a common phenomenon in adolescence, affecting almost all young people, whether as perpetrators or victims [5]. Another defining characteristic of rule-breaking behavior in adolescence is its occurrence in groups, frequently within the context of friendship circles [5]. Furthermore, numerous studies have demonstrated that contact with rule-breaking friends is a significant predictor of rule-breaking behavior [6,7,8,9]. Therefore, when explaining rule-breaking behavior in adolescence, it is important to consider whether it is committed alone or together with friends. Nevertheless, this perspective has yet to be empirically investigated in the context of moral emotions.

### 1.1. Situational Action Theory

SAT proposes that rule-breaking behavior is the result of a situational perception–choice process. The perception–choice process is based on the interaction between an individual’s propensity to engage in rule-breaking and the characteristics of the environment/situation that promote or inhibit rule-breaking. Within the perception–choice process, action alternatives are evaluated through the so-called moral filter. As a result, rule-breaking behavior is either evaluated as an appropriate action alternative (failure of the moral filter) or it is not. If the moral filter fails, control mechanisms (internal: self-control; and external: deterrence) regulate behavior [2]. Rule-breaking behavior is the result of the perception–choice process under the two following conditions. First, rule-breaking is evaluated as an appropriate action alternative, which primarily depends on one’s moral standards (the person’s knowledge and the internalization of moral norms [10]). Second, one fails to act in accordance with one’s individual moral standards, e.g., due to low self-control [2].

### 1.2. Individual Propensity to Engage in Rule-Breaking Behavior

#### 1.2.1. Moral Emotions

Moral emotions are valid indicators of one’s moral standards and are thought to act as a link between moral standards and behavioral outcomes [10]. Theory distinguishes between (a) morally relevant other-oriented emotional processes (e.g., sympathy) and (b) morally relevant self-conscious emotions [11]. This study focuses on self-conscious moral emotions, which will be referred to as moral emotions. Negatively valenced moral emotions, such as guilt and shame, arise when there is a discrepancy between one’s moral standards and one’s behavior or thoughts [10]. Feelings of guilt often lead individuals to engage in positive actions such as confessions and apologies [10,12,13], while also acting as a deterrent to rule-breaking behavior [14,15,16]. Shame is positively associated with prosocial behavior, but it is also positively correlated with negative behaviors such as blaming others [17,18,19]. Therefore, its influence on deterring rule-breaking behavior is not as pronounced as that of guilt [15].

Moral emotions either arise as a direct consequence of behavior or are anticipated as part of the decision-making process [10]. Anticipated moral emotions are often measured in hypothetical situations. One test for assessing guilt and shame proneness (general tendency to feel guilty/ashamed [10]) is the Test of Self-Conscious Affect-Adolescent (TOSCA-A [20]). It measures the anticipated affective responses to predefined behaviors in a variety of situations. Another type of hypothetical situation often used to measure anticipated moral emotions is a moral dilemma. A moral dilemma describes a moral conflict with two incompatible alternative actions, both of which can be morally justified [21]. Participants are asked to describe what they would do or decide between two given alternative actions. Moral emotions are then elicited for the chosen or both alternatives.

#### 1.2.2. Self-Control

The role of self-control in explaining rule-breaking behavior has been highlighted, for example, in the General Theory of Crime/Self-Control Theory [22], and is empirically supported, as shown in a meta-analysis conducted by Vazsonyi et al. [23]. In SAT, self-control is defined as follows: (1) the general ability to exercise self-control, which “is the capacity to act in accordance with one’s own personal morality when externally pressurized to act otherwise” and (2) the situational ability to exercise self-control, meaning to “withstand external pressure to act against one’s own personal morality” [2] (p. 268). The former is part of the individual’s propensity to engage in rule-breaking, and the latter is part of the perception–choice process.

SAT posits that self-control only affects rule-breaking behavior when it is evaluated as an appropriate action alternative (moral filter failure). Most previous research, e.g., [3,24], seems to confirm this assumption. It shows that the effect of self-control on rule-breaking behavior depends on the level of morality. Self-control contributes to explaining rule-breaking behavior only among adolescents with “weak morality” (indicated by low levels of moral rules and shame [3] or moral values [24]).

### 1.3. Unstructured Socializing as an Situational Factor That Promotes Rule-Breaking Behavior

Morality appears to moderate not only the effect of self-control on rule-breaking behavior, but also the effect of situational factors that facilitate rule-breaking. Results by Gerstner and Oberwittler’s [25] findings suggest that the relation between unstructured socializing and rule-breaking behavior is significantly stronger for adolescents with weak moral beliefs and low self-control than for adolescents with strong moral beliefs and high self-control. The term “unstructured socializing” was introduced within the ‘routine activity approach’ [26] and the work of Osgood et al. [27]. It describes unsupervised (e.g., without parental control) activities spent with peers doing ‘nothing particular’. These activities appear to be particularly conducive to rule-breaking behavior because the presence of peers makes rule-breaking easier and more rewarding, being unsupervised leads to less social control, and the lack of structure provides time for rule-breaking [27]. In line with this, Weerman et al. [28] showed that activities with friends characterized by at least two of the following aspects are positively associated with rule-breaking behavior: 1. just socializing; 2. being in public; and 3. being unsupervised.

### 1.4. Friends’ Influence on Adolescents’ Rule-Breaking Behavior

It is well known that rule-breaking peers/friends promote rule-breaking behavior, e.g., [24,25,26,27,28,29]. Social learning theory [30] suggests that the likelihood of breaking rules is increased by spending more time with others who exhibit rule-breaking behavior than with people who behave in a rule-compliant manner. Others who break rules serve as role models, which leads to rule-breaking behavior being justified and defined as desirable. Within these social interactions, rule-breaking behavior is rewarded and reinforced, for example, by sharing positive stories about rule-breaking, gestures of appreciation, laughter, or positive verbal feedback [24]. A meta-analysis conducted by Pratt et al. [31] showed fairly strong empirical support for social interactions influencing one’s attitudes and evaluations of rule-breaking behaviors, which is one of the core propositions of social learning theory. However, the assumption that rule-breaking behavior is reinforced by imitation and rewards from a rule-breaking social environment showed only moderate effects, if any.

According to social identity theory [32,33], a person’s self-concept consists of two distinct parts—personal identity and social identity. Social identity is based on the categorizations of a social group (e.g., rule-breaking friends). An important point within this theory is that group conforming behavior does not result from social pressure, but rather from the integration of parts of the social identity into the personal identity. That is, if breaking rules is part of the identity of the peer group, and is therefore defined as desirable within the group, group members will integrate this definition into their personal identity and behave accordingly by engaging in rule-breaking behavior.

### 1.5. The Present Study

Given that rule-breaking in adolescence tends to occur within a group dynamic [5], it is reasonable to assume that adolescents primarily consider rule-breaking with friends when assessing their own rule-breaking behavior. However, it is also plausible to assume that adolescents evaluate rule-breaking behavior from different perspectives, influenced by whether they are asked to evaluate their own rule-breaking or that committed together with friends. This results in the aim of the present study. It will be examined whether the interactions of moral emotions with (1) self-control and (2) unstructured socializing in explaining rule-breaking behavior differ depending on whether adolescents evaluate their own rule-breaking or that committed together with friends. Furthermore, three operationalizations of moral emotions are distinguished: (1) guilt; (2) shame-proneness; and (3) anticipated emotions in moral conflicts (AEMC). The first two can be interpreted as situation-independent and more trait-related operationalizations of moral emotions, whereas the third is more situation-specific.

This leads to the following research questions. The first two deal with the interaction between moral emotions and self-control in predicting rule-breaking behavior (own and with friends). The third and fourth questions relate to the interaction between moral emotions and unstructured socializing in predicting rule-breaking behavior (own and with friends).

1. Do moral emotions (a. guilt- and b. shame proneness as well as c. AEMC) interact with self-control in explaining own rule-breaking behavior?
2. Do moral emotions (a. guilt- and b. shame proneness as well as c. AEMC) interact with self-control in explaining rule-breaking with friends?
3. Do moral emotions (a. guilt- and b. shame proneness as well as c. AEMC) interact with unstructured socializing in explaining own rule-breaking behavior?
4. Do moral emotions (a. guilt- and b. shame proneness as well as c. AEMC) interact with unstructured socializing in explaining rule-breaking with friends?

It is assumed that previous findings on the interaction between morality and self-control [3] and unstructured socializing [19] will be replicated in explaining one’s own rule-breaking behavior. Assumptions for explaining rule-breaking with friends were formulated against the background of the exploratory approach of this study.

Given that moral emotions seem to weaken the effect of rule-breaking peers on rule-breaking behavior [4], it can be hypothesized that the presence of friends does not make a relevant difference. Thus, the interactions would be the same as when explaining one’s own rule-breaking behavior. On the other hand, it could be assumed that the presence of friends leads to diffusion of responsibility, i.e., the responsibility for rule-breaking behavior is shared among all the individuals present, which weakens the individual’s sense of responsibility. Thus, environmental factors, such as unstructured socializing with friends, may become more important, and individual factors, such as moral emotions, may become less important for rule-breaking with friends than for own rule-breaking. This would mean that the moderating effect of moral emotions is not evident in explaining rule-breaking behavior.

## 2. Materials and Methods

### 2.1. Participants and Procedure

Participants were recruited in Germany and Switzerland. As the data collection took place during the coronavirus pandemic and it was therefore to be expected that the positive responses would be lower, different schools in Germany (Hamburg and North Rhine-Westphalia) attended by adolescents aged between 12 and 18 were contacted. Care was taken to ensure that the school types and districts were as balanced as possible. In addition, part of the data collection was carried out as part of an evaluation project conducted in secondary schools in Germany and Switzerland (German-speaking).

Data were collected using a series of self-report questionnaires that could be completed online or in a paper–pencil version. Demographic differences between adolescents who answered the online version and those who answered the paper–pencil version were predominantly small [34], which is why data collected from the online and the paper-pencil versions were analyzed together.

Data collection took place in class with at least one trained test administrator (project staff or psychology students) present. Inclusion criteria were to be between 12 and 18 years old and have a reasonable understanding of German. A total of *n* = 323 adolescents took part in the study, of which *n* = 154 had to be excluded because they did not complete the questionnaire (*n* = 133) or did not fit into the target age group (*n* = 21). It was examined whether adolescents with certain socio-demographic characteristics systematically dropped out or were excluded. Inclusion/exclusion was exclusively related to gender (χ^2^ (1) = 7.78, *p* = 0.005). As this was a small effect (*V* = 0.18, *p* = 0.005) [34] and there were no associations with other socio-demographic characteristics, it is assumed that this did not bias the results.

Single missing data were imputed using multiple imputation (fully conditional specification), resulting in five imputed datasets. Little’s Missing Completely at Random (MCAR) test was run before data imputation showing an MCAR pattern of missing data (χ^2^ (16,913) = 14,449.28, *p* = 0.998). Parameter estimates were averaged/pooled for point estimation (a combined score). The number of missing data ranged from 4 (2%) to 48 (28%) cases per scale (see Table A1 for the *N* of each scale in the original data). Spiess and Goebel [35] showed that reliable results are possible, even from datasets in which 65% of the data had to be imputed.

For sample characteristics see Table 1. The final sample comprised *N* = 169 adolescents (54% female) with a mean age of 14.95 years (*SD* = 1.7; range = 12–18 years). Just over half of the adolescents (*n* = 95, 56%) reported attending grammar school (dt. ‘Gymansium’), which is a specific type of secondary school in Germany and Switzerland. Most of the adolescents were born in Germany/Switzerland (*n* = 158, 93%) and mainly spoke German at home (*n* = 121, 72%). Informed consent was given by adolescents 16 years or older, and parental informed consent was obtained for adolescents younger than 16 years. Participation was voluntary and could be refused or discontinued without disadvantage for the participating adolescents. The study was approved by the school authorities of the respective federal state.

### 2.2. Measures

#### 2.2.1. Guilt- and Shame-Proneness

Adolescents’ guilt- and shame-proneness were measured using the Test of Self-Conscious Affect-Adolescent (TOSCA-A) [20]. As the TOSCA-A has not yet been validated for German-speaking countries and no similar German-language test was available, it was translated into German using a forward–backward procedure. The TOSCA-A consists of 10 negative (e.g., ‘You trip in the cafeteria and spill your friend’s drink.’) and 5 positive hypothetical situations. For each situation, an affective response is presented for guilt, shame, detachment, and externalization and pride. Only the subscales for guilt (e.g., ‘I would feel very sorry. I should have watched where I was going.’) and shame (e.g., ‘I would be thinking that everyone is watching me and laughing.’) were used. Results on the factor structure and test quality of the TOSCA-A indicate a two-factor structure for the guilt and shame subscales, as well as their convergent validity, discriminant validity, and metric invariance [36]. On a 5-point Likert scale (1 = very unlikely to 5 = very likely), adolescents rated how likely they were to experience reactions of guilt and shame. Higher scores indicate higher levels of guilt- and shame-proneness. Internal consistency in the present sample was α = 0.85 for the guilt scale and α = 0.88 for the shame scale. In the following, the variables will be referred to as *guilt* and *shame*.

#### 2.2.2. Anticipated Emotions in Moral Conflicts

Adolescents’ anticipated emotions were measured used the Questionnaire for the Assessment of Moral Attitudes in Adolescence (QAMA-A; original title: Fragebogen zur Erfassung moralischer Einstellung im Jugendalter, FEME-J; [37]) in its third and shortened version. It consists of six moral conflicts presented in pictures and text. The conflicts are based on the idea of Weller and Lagattuta [38]. They have been adapted to situations that adolescents are confronted with in their everyday life and modified according to recommendations of Christensen and Gomila [21]. The situations include moral- and law-relevant rule-breaking (e.g., lying and stealing). Female adolescents completed the ‘female version’ of the questionnaire, in which all protagonists were female. In the ‘male version’, protagonists were male. This means that emotions were anticipated in ingroup-situations (same sex) only. For each of the six situations, two action alternatives were given (selfless vs. selfish) and the adolescents chose one of them. Intensity and valence of emotions were rated for both selfless decisions (e.g., ‘Imagine that you decided not to take the ticket. How good or bad do you feel about that?’) and selfish decisions (e.g., ‘Imagine you’ve decided to take the ticket. How good or bad do you feel about that?’). Emotions anticipated in the case of rule-breaking (selfish decision) were included in the analysis. Responses were given on a 5-point Likert scale (1 = very good, 3 = neither good nor bad, 5 = very bad). Low scores indicate positively valenced moral emotions (e.g., moral pride), medium scores indicate mixed/neutral emotions, and high scores indicate negatively valenced moral emotions (e.g., guilt). The internal consistency in the present sample was α = 0.69. Further results on test quality are not yet available. In the following, the variable is called *AEMC*.

#### 2.2.3. Self-Control

Adolescents’ self-control was measured with 15 items of a newly developed questionnaire called School-related Personality and Resources in Grades 5 to 10 (SPR 5–10; original title: Schulbezogene Persönlichkeitsmerkmale und Ressourcen in den Klassenstufen 5 bis 10; [39]). In selecting items, care was taken to ensure that the content of the items was also appropriate for adolescents over the age of 16. Items from the following subscales of the SPR 5–10 were used: (1) delay of gratification (e.g., ‘I find it hard to budget my money.’), (2) frustration tolerance (e.g., ‘I cannot stand being overruled.’), (3) self-regulation (e.g., ‘I find it easy to work on a task even when people sitting next to me are talking.’), and (4) self-control (e.g., ‘Sometimes things slip out that I didn’t mean to say.’). Responses were given on a 5-point Likert scale ranging from 1 = not at all true to 5 = completely true. The items used are negatively worded, so higher scores indicate lower levels of self-control and therefore a higher propensity to engage in rule-breaking behavior. The scores of all four scales were summed, resulting in an internal consistency of α = 0.71 for the present sample. Further results on test quality are not yet available. The variable is referred to below as *low self-control*, as low levels of self-control are a risk factor for rule-breaking behavior.

#### 2.2.4. Unstructured Socializing

Unstructured socializing was measured with four items (e.g., ‘How often have you spent time outside with your friends in the evening?’), which were based on the items developed by Osgood et al. [27]. As the items were developed by Osgood et al. [27] in the 1990s, they were revised to make them more relevant to the current era. Responses were given on a 5-point Likert scale (1 = never to 5 = always). Higher scores indicate more frequent unstructured socializing. The internal consistency was α = 0.78 in the present sample. The variable is referred to as *unstructured socializing* below.

#### 2.2.5. Own Rule-Breaking Behavior

To measure adolescents’ own rule-breaking behavior, the *Rule-breaking Behavior* (e.g., ‘I drink alcohol without my parents’ permission.’) and *Dissocial Behavior* (e.g., ‘I physically attack others.’) scales from the Youth-Self Report section of the German version of the Child Behavior Checklist (CBCL, YSR 11-18R) [40,41] were used. Responses were given on a 3-point Likert scale (1 = not true, 2 = somewhat or sometimes true, 3 = very or often true). Higher scores indicate more frequent own rule-breaking behavior. The scores of both scales were summed (α = 0.93 in present sample). In the following, the variable will be referred to as *own rule-breaking*.

#### 2.2.6. Rule-Breaking with Friends

Rule-breaking with friends was measured with 13 items based on the Youth Self-Report 11-18R of the Child Behavior Checklist (CBCL, YSR 11-18R) [40,41]. Items from the Rule-breaking Behavior and Dissocial Behavior scales were modified by asking about rule-breaking behavior that is explicitly committed together with friends (e.g., ‘When you are with your friends, you [German translation: ‘ihr’, which means you and your friends] destroy things that belong to others.’). Responses were given on a 3-point Likert scale (1 = not true, 2 = somewhat or sometimes true, 3 = very or often true. Higher scores indicate more frequent rule-breaking with friends. The scores of both scales were summed (α = 0.91 in the present sample). In the following, the variable will be referred to as *rule-breaking with friends*.

### 2.3. Data Analytic Strategy

Statistical analyses were performed using IBM SPSS Statistics (version 29.0). First, descriptive and correlational analyses were run. Assumptions for linear regression analyses were then tested. Linearity was met for relations between predictors and outcome variables, but not for relations between moderators and predictors. Normality was met for all variables except for a slight deviation for the outcome variable. Homoscedasticity and independence were met. Hypotheses were tested by calculating four hierarchical linear regression analyses—one for each research question. Variables were mean-centered prior to inclusion in multiplicative interaction terms. Predictor (low self-control and unstructured socializing) and moderator (guilt, shame, and AEMC) variables were added in the first block (model 1), followed by interaction terms in the second block (model 2) and control variables (gender and sex) in the third block (model 3). Significant interactions were plotted. For this purpose, the continuous variables *guilt*, *shame*, and *AEMC* were z-standardized and recoded into categorical variables with low (−0.5 SD), medium (M), and high (+0.5 SD) levels. The significance level of the analyses was determined as α = 0.05. Effect sizes were interpreted according to Cohen’s [34] recommendations: small effect (*R*^2^ = 0.02), moderate effect (*R*^2^ = 0.13), and large effects (*R*^2^ = 0.26).

## 3. Results

### 3.1. Descriptive and Correlational Analysis

The results of the descriptive analyses are shown in Table A1. Correlational analyses (Table 2) show that *guilt* (*r* = −0.23, *p* = 0.003), *AEMC* (*r* = −0.33, *p* < 0.001), *low self-control* (*r* = 0.50, *p* < 0.001), and *unstructured socializing* (*r* = 0.55, *p* < 0.001) were related to *own rule-breaking*. Directions of these relations indicate that the higher the levels of guilt-proneness and AEMC, the lower the frequency of own rule-breaking behavior. Furthermore, the lower the levels of self-control, and the more frequent the unstructured socializing, the higher the frequency of own rule-breaking behavior.

The relationships of *rule-breaking with friends* were relatively similar, where *AEMC* (*r* = −0.25, *p* < 0.001), *low self-control* (*r* = 0.33, *p* < 0.001), and *unstructured socializing* (*r* = 0.47, *p* < 0.001) were related to it, but *guilt* (*r* = −0.14, *p* = 0.075) was not. The directions of these relations suggest that the higher the levels of AEMC, the less frequent the rule-breaking with friends. Furthermore, the lower the levels of self-control, and the more frequent the unstructured socializing, and the more frequent the rule-breaking behavior with friends.

*Shame* was not related to either *own rule-breaking* (*r* = 0.08, *p* = 0.301) or *rule-breaking with friends* (*r* = 0.07, *p* = 0.393). *Low self-control* was related to *guilt* (*r* = −0.27, *p* < 0.001) and *AEMC* (*r* = −0.24, *p* = 0.002), but not to *shame* (*r* = −0.08, *p* = 0.324). *Unstructured socializing* was not related to *guilt* (*r* = −0.13, *p* = 0.092), *shame* (*r* = −0.14, *p* = 0.048), nor *AEMC* (*r* = −0.11, *p* = 0.177).

### 3.2. Interaction between Self-Control and Moral Emotions

The results of the first regression analysis testing the interaction between self-control and moral emotions in explaining *own rule-breaking behavior* were as follows (for details, see Table 3). Model 1 was significant, *F*(4, 164) = 20.88, *p* < 0.001, explaining 34% of the variance (large effect; [34]) in *own rule-breaking*. Model 2 (including the interaction terms) was also significant, *F*(7, 161) = 14.19, *p* < 0.001, explaining 38% of the variance (large effect; [34]) in *own rule-breaking*, with a significant change in *F* (*p* = 0.012). The interaction between *low self-control* and *AEMC* was significant (*p* = 0.002), whereas the interactions between *low self-control* and *guilt* (*p* = 0.344), and *low self-control* and *shame* (*p* = 0.580) were not.

**Figure 1:**
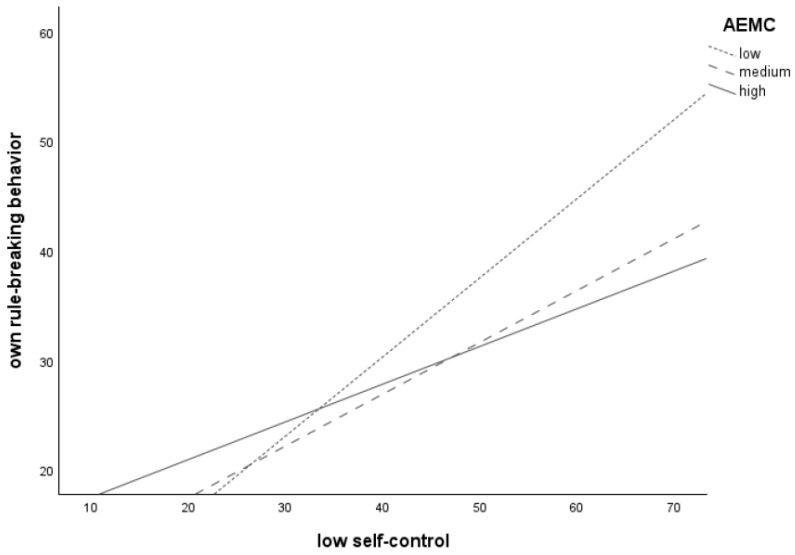
Relation between low self-control and own rule-breaking behavior for adolescents with low (*n* = 57), medium (*n* = 52), and high (*n* = 60) levels of anticipated emotions in moral conflicts (AEMC).

The plotting of the significant interaction between *low self-control* and *AEMC* (see Figure 1) revealed that higher levels of *low self-control* were associated with more frequent *own rule-breaking* for adolescents with low (*n* = 57), medium (*n* = 52), and high (*n* = 60) levels of *AEMC*, as all three lines increased. The line for the low AEMC group was the steepest, followed by the medium AEMC group. The line for the high AEMC group was the flattest. This suggests that the relation between self-control and one’s own rule-breaking behavior is at its strongest at low levels of *AEMC*.

The results of the second regression analysis testing the interaction between self-control and moral emotions in explaining *rule-breaking with friends* were as follows (for details see Table 3). Model 1 was significant, *F*(4, 164) = 8.31, *p* < 0.001, explaining 17% of the variance (moderate effect; [34]) in *rule-breaking with friends*. Model 2 (including the interaction terms) was also significant, *F*(7, 161) = 5.50, *p* < 0.001, explaining 19% of the variance (moderate effect; [34]) in *rule-breaking with friends*, but without a significant change in *F* (*p* = 0.196). Furthermore, none of the interaction terms were significant (low self-control * guilt: *p* = 0.613, low self-control * shame: *p* = 0.896), with the interaction between *low self-control* and *AEMC* just missing significance at *p* = 0.053.

### 3.3. Interaction between Unstructured Socializing and Moral Emotions

The results of the third regression analysis testing the interaction between unstructured socializing and moral emotions in explaining own rule-breaking behavior were as follows (for details see Table 4). Model 1 was significant, *F*(4, 164) = 34.49, *p* < 0.001, explaining 46% of the variance (large effect; [34]) in *own rule-breaking*. Model 2 (including the interaction terms) was also significant, *F*(7, 161) = 25.54, *p* < 0.001, explaining 53% of the variance (large effect; [34]) in *own rule-breaking*, with a significant change in *F* (*p* < 0.001). The interactions between *unstructured socializing* and *guilt* (*p* < 0.001) and between *unstructured socializing* and *shame* (*p* < 0.001) were significant, whereas the interaction between *unstructured socializing* and *AMEC* was not (*p* = 0.274).

**Figure 2:**
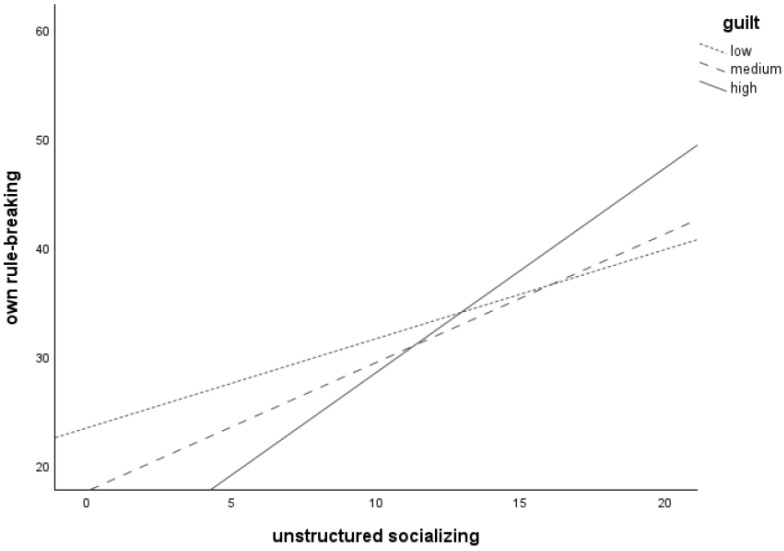
Relation between unstructured socializing and own rule-breaking behavior for adolescents with low (*n* = 46), medium (*n* = 76), and high (*n* = 47) levels of guilt-proneness.

Plotting the significant interaction between *unstructured socializing* and *guilt* (see Figure 2) revealed that more unstructured socializing is associated with more frequent *own rule-breaking* for adolescents with low (*n* = 46), medium (*n* = 76), and high (*n* = 47) levels of *guilt*, as all three lines increase. The line for the high guilt group was the steepest, followed by the medium guilt group. The line for the low guilt group was the flattest. This suggests that the relation between *unstructured socializing* and one’s own rule-breaking behavior is strongest at high levels of *guilt*.

**Figure 3:**
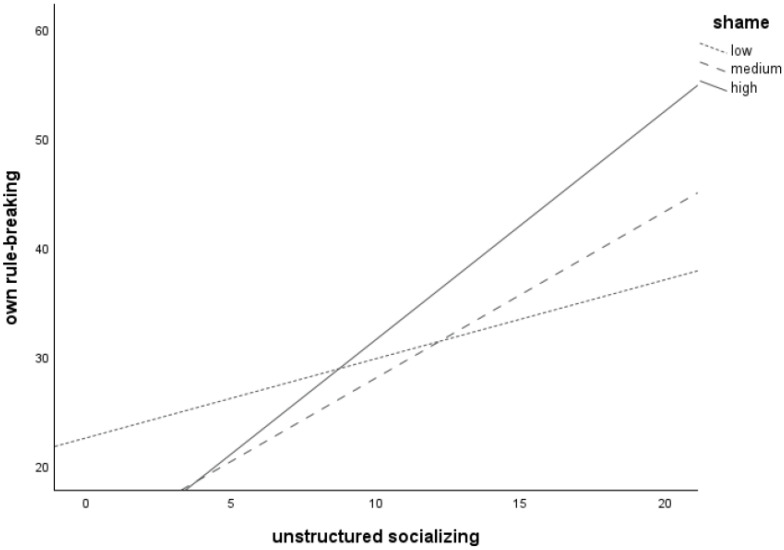
Relation between unstructured socializing and own rule-breaking behavior for adolescents with low (*n* = 56), medium (*n* = 65), and high (*n* = 48) levels of shame-proneness.

Plotting of the significant interaction between *unstructured socializing* and *shame* (see Figure 3) revealed that more unstructured socializing is associated with more frequent *own rule-breaking* for adolescents with low (*n* = 56), medium (*n* = 65), and high (*n* = 48) levels of *shame*, as all three lines increase. The line for the high shame group was the steepest, followed by the medium shame group. The line for the low shame group was the flattest. This suggests that the relation between *unstructured socializing* and one’s own rule-breaking behavior is strongest at high levels of *shame*.

The findings of the fourth regression analysis testing the interaction between unstructured socializing and moral emotions in explaining *rule-breaking with friends* were as follows (for details see Table 4). Model 1 was significant, *F*(4, 164) = 18.42, *p* < 0.001, explaining 31% of the variance (large effect; [33]) in *rule-breaking with friends*. Model 2 (including the interaction terms) was also significant, *F*(7, 161) = 15.21, *p* < 0.001, explaining 40% of the variance (large effect; [34]) in *rule-breaking with friends*, with a significant change in *F* (*p* < 0.001). Interactions between unstructured socializing and guilt (*p* < 0.001) as well as between unstructured socializing and shame (*p* < 0.001) were significant, whereas the interaction between unstructured socializing and AMEC was not (*p* = 0.086).

**Figure 4:**
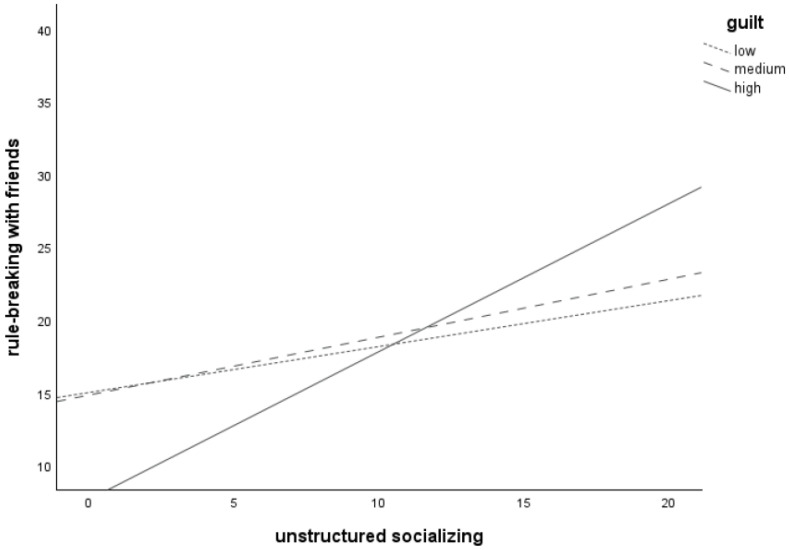
Relation between unstructured socializing and rule-breaking behavior committed together with friends for adolescents with low (*n* = 46), medium (*n* = 76), and high (*n* = 47) levels of guilt-proneness.

Plotting of the significant interaction between *unstructured socializing* and *guilt* (see Figure 4) revealed that more unstructured socializing is associated with more frequent *rule-breaking with friends* for adolescents with low (*n* = 46), medium (*n* = 76), and high (*n* = 47) levels of *guilt*, as all three lines increase. The line for the high-guilt group was the steepest, followed by the medium-guilt group. The line for the low-guilt group was the flattest. This suggests that the relation between *unstructured socializing* and *rule-breaking with friends* is strongest at high levels of *guilt*.

**Figure 5:**
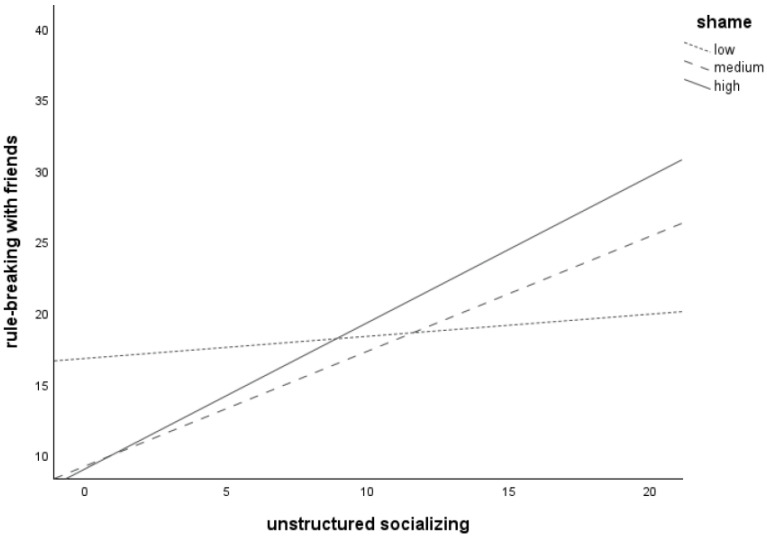
Relation between unstructured socializing and rule-breaking behavior committed together with friends for adolescents with low (*n* = 56), medium (*n* = 65), and high (*n* = 48) levels of shame-proneness.

Plotting of the significant interaction between *unstructured socializing* and *shame* (see Figure 5) revealed that more unstructured socializing is associated with more frequent *rule-breaking with friends* for adolescents with low (*n* = 56), medium (*n* = 65), and high (*n* = 48) levels of *shame*, as all three lines increase. The line for the high shame group was the steepest, followed by the medium shame group. The line for the low shame group was the flattest. This suggests that the relation between *unstructured socializing* and *rule-breaking with friends* is strongest at high levels of *shame*.

After the inclusion of the control variables (gender and age) in all regression analyses in Model 3, the results of the interactions remained unchanged.

## 4. Discussion

To our knowledge, the present study was the first to examine whether moral emotions (guilt-, shame-proneness, and anticipated emotions in moral conflicts (AEMC)) interact with self-control and unstructured socializing in explaining rule-breaking with friends. Results showed that some of the interactions tested differed depending on whether adolescents rated their own rule-breaking behavior or rule-breaking with friends. Anticipated emotions in moral conflicts (AEMC) interacted with self-control in explaining own rule-breaking, whereas guilt- and shame-proneness did not. The relationship between self-control and own rule-breaking behavior was the strongest for adolescents with low levels of AEMC and weakest for adolescents with high levels of AEMC. This suggests that AEMC ‘buffer’ the effect of low self-control in promoting own rule-breaking behavior. In explaining rule-breaking with friends moral emotions—neither guilt-, or shame-proneness, nor AEMC—did not interact with self-control.

The association between unstructured socializing and rule-breaking behavior (own and with friends) was moderated by guilt- and shame-proneness, but not by AMEC. The relation between unstructured socializing and rule-breaking was the strongest among adolescents prone to guilt and shame. This suggests that high levels of guilt- and shame-proneness do not buffer the effect of unstructured socializing in explaining rule-breaking behavior, but rather strengthen it.

### 4.1. Interaction between Self-Control and Moral Emotions

The interaction between self-control and AEMC in explaining adolescents’ own rule-breaking behavior is in line with previous findings (for an overview, see Pauwels et al. [42]), who suggest that self-control is primarily relevant in explaining rule-breaking behavior among adolescents with low levels of morality. In contrast, AEMC and self-control did not interact in explaining rule-breaking with friends but were independently related to it. According to this, either the relevance of moral emotions decreases or that of self-control increases when adolescents report rule-breaking with friends.

The latter is consistent with the SAT definitions of self-control (trait and situational), which emphasize that self-control is particularly important when a person is led by others to act inconsistently with personal morality [2]. Furthermore, while self-control and moral emotions interact in most previous studies, there are also studies in which the interaction between morality and self-control does not (partially) explain rule-breaking behavior. For example, Gallupe and Baron [43] found no interaction between moral values and self-control in explaining hard and soft drug use among adolescents. In addition, moral judgments appear to have moderated the effect of self-control only in explaining a combined delinquency score (including different types of delinquency) and not in explaining violent and property offending separately [44]. Furthermore, the interaction between moral judgments and self-control could not be confirmed in longitudinal analyses [45]. These and the results of the present study suggest that the interaction between morality and self-control does not contribute to the explanation of rule-breaking behavior per se but depends on the operationalization of rule-breaking behavior. Therefore, further research is needed on the ‘general validity’ of the interaction between morality and self-control in explaining rule-breaking behavior.

The fact that self-control is moderated by AEMC in explaining one’s own rule-breaking behavior, and not by guilt- and shame-proneness, may be explained by the following. In the present study, AEMC was measured in situations involving moral rule-breaking (helping and lying) and law-relevant rule-breaking (stealing and physically assault). This means that adolescents anticipated their emotions for behavior that was relatively similar to the behavior in the items measuring rule-breaking behavior. In contrast, the situations in which guilt- and shame-proneness were measured primarily involved moral rule-breaking and not law-relevant rule-breaking.

### 4.2. Interaction between Unstructured Socializing and Moral Emotions

In the present study, unstructured socializing and moral emotions (guilt- and shame-proneness) interact in the explanation of one’s own and rule with friends. That is in line with the results by Gerstner and Oberwittler [25], which show that moral values (as a combined score with self-control) interact with unstructured socializing in explaining rule-breaking behavior. Yet, the present results on rule-breaking with friends differ in one important respect—strong morality did not weaken the effect of unstructured socializing but fostered it. In the case of shame, this finding can be partially explained by previous research showing that shame can also lead to destructive, avoidant behavior [17,18,19].

It can be reasonably assumed that when adolescents must decide whether to break rules with friends, levels of guilt and shame are likely to be related to both—breaking rules and not conforming to the group by not breaking rules. Unstructured socializing, as a situation that favors rule-breaking behavior, is likely to make it difficult not to act in a group-conforming way. Therefore, the fear that nonconforming behavior will not be rewarded or even punished by friends’ reactions may increase the pressure to act in accordance with friends’ expectations. As a result, the relationship between unstructured socializing and rule-breaking behavior is stronger for guilt- and shame-prone adolescents.

The findings reported by Hoeben et al. [29] demonstrate the importance of examining the mediators and moderators of the relationship between unstructured socializing and rule-breaking behavior. The present results support this statement. Future research should examine the conditions under which the relationship between unstructured leisure activities and rule-breaking behavior is moderated by moral emotions and, in particular, whether they weaken or, as in the present study, strengthen the effect.

## 5. Limitations and Conclusions

The following limitations must be considered when interpreting the results of the present study. First, the underlying sample was not fully representative, e.g., in terms of adolescents’ educational background, which limits the generalizability of the results. In addition, the relatively small sample size of the present study (*N* = 169) may have led to missing significant effects. The relatively high dropout rate can be explained by the fact that, in at least two classes, data collection was abandoned due to spontaneous interruptions in school. In addition, a dropout rate of 30% appears to be average for online studies [46]. For this reason, the results should be verified in a more representative and larger sample.

For following research, it is important to consider the choice of instruments used to assess the rule-breaking behavior that is committed together with friends. It is important to stress that the scale used in the present study is an adapted version that has not yet been validated. Nevertheless, the internal consistency of the scale was quite good. Furthermore, there seems to be a tendency to overestimate when reporting friends’ rule-breaking behavior. The perception of friends’ behavior seems to correlate with one’s own behavior [25]. Essentially, when adolescents frequently break rules, they tend to evaluate their friends’ behavior in a similar way. It is important for future research to explore whether this pattern extends to rule-breaking behavior committed together with friends.

Furthermore, it is important to highlight that AEMC were measured exclusively in same-sex situations. In subsequent research, it is essential to test for measurement invariance as well as to measure AMEC in outgroup situations, if possible. For childhood, the results from Weller and Lagattuta [38] demonstrate that anticipated emotions differ depending on whether they relate to ingroup or outgroup situations.

Taken together, the current study suggests that the interactions of moral emotions in explaining rule-breaking behavior differ (a) depending on whether adolescents report their own rule-breaking behavior or that committed together with friends and (b) depending on the operationalization of moral emotions used. The former emphasizes the necessity for the inclusion of friends and peers in the measurement of rule-breaking behavior in adolescence. The latter suggests that moral emotions do not protect against rule-breaking behavior per se. This is important, not least for prevention and intervention programs that focus on moral emotions. Given the methodological limitations and the fact that this was the first study to empirically test the moderating effect of moral emotions in explaining rule-breaking with friends, the results should be interpreted as exploratory. Nevertheless, they provide a good starting point for further research, the relevance of which is demonstrated by the present work.

## Figures and Tables

**Table 1:** Sample characteristics (*N* = 169).

Sex	Female: 54%	Male: 46%
Age (in years)	*M* = 14.95, *SD* = 1.7 *Min* = 12, *Max* = 18	
School type	Grammar school: 56%	Others: 44%
Birth country	Germany/Switzerland: 93%	Others: 7%
Language spoken at home	German: 72%	Others: 28%

**Table 2:** Bivariate correlations between study variables.

	(1)	(2)	(3)	(4)	(5)	(6)	(7)	(8)	(9)
(1) Age	1	−0.16 (0.039)	−0.18 (0.020)	−0.21 (0.006)	−0.19 (0.014)	0.12 (0.116)	0.39 (<0.001)	0.151 (0.051)	0.22 (0.004)
(2) Gender	−0.16 (0.039)	1	0.13 (0.094)	0.28 (<0.001)	0.19 (0.014)	−0.09 (0.256)	−0.04 (0.582)	−0.07 (0.362)	0.005 (0.948)
(3) Guilt	1	-	-	-	-	-	-	-	-
(4) Shame	0.59 (<0.001)	1	-	-	-	-	-	-	-
(5) Anticipated emotions in moral conflicts (AEMC)	0.63 (<0.001)	0.32 (<0.001)	1	-	-	-	-	-	-
(6) Low self-control	−0.27 (<0.001)	−0.08 (0.324)	−0.24 (0.002)	1	-	-	-	-	-
(7) Unstructured socializing	−0.13 (0.092)	−0.14 (0.080)	−0.11 (0.177)	0.32 (<0.001)	1	-	-	-	-
(8) Own rule-breaking	−0.23 (0.003)	0.08 (0.301)	−0.33 (<0.001)	0.50 (<0.001)	0.55 (<0.001)	1	-	-	-
(9) Rule-breaking w. friends	−0.14 (0.075)	0.07 (0.393)	−0.25 (<0.001)	0.33 (<0.001)	0.47 (<0.001)	0.812 (<0.001)	1	-	-

Notes. two-tailed; *p*-values in parentheses; gender is coded as 0 = males, 1 = females; high levels of guilt indicate strong guilt-proneness; high levels of shame indicate strong shame-proneness; high levels of AEMC indicate feeling bad with selfish decisions; high levels of low self-control indicate low self-control; high levels of unstructured socializing indicate frequent unstructured leisure activities with friends; high levels of own rule-breaking indicate frequent rule-breaking behavior adolescents themselves actively show; high levels of rule-breaking with friends indicate frequent rule-breaking with friends.

**Table 3:** Interaction between low self-control and moral emotions in explaining rule-breaking behavior (own and with friends).

*N* = 169	Model 1				Model 2				Model 3			
	*B*	95% *CI*	*SE*(*B*)	*p*	*B*	95% *CI*	*SE*(*B*)	*p*	*B*	95% *CI*	*SE*(*B*)	*p*
Own rule-breaking												
Low self-control	0.45	0.31–0.58	0.07	<0.001	0.49	0.35–0.62	0.07	<0.001	0.48	0.34–0.62	0.07	<0.001
Guilt	−0.09	−0.24–0.06	0.07	0.270	−0.09	−0.24–0.06	0.08	0.226	−0.10	−0.25–0.05	0.08	0.192
Shame	0.18	0.07–0.29	0.06	0.001	0.17	0.06–0.28	0.06	0.003	0.19	0.07–0.30	0.06	0.002
AEMC	−0.47	−0.79–−0.16	0.16	0.003	−0.42	−0.73–−0.11	0.16	0.008	−0.39	−0.70–−0.07	0.16	0.017
Low self-control × guilt	–	–	–	–	0.01	−0.01–0.02	0.01	0.344	0.01	−0.01–0.02	0.01	0.359
Low self-control × shame	–	–	–	–	0.00	−0.01–0.02	0.01	0.580	0.00	−0.01–0.02	0.01	0.598
Low self-control × AEMC	–	–	–	–	−0.06	−0.10–−0.02	0.02	0.002	−0.06	−0.10–−0.02	0.02	0.003
Gender	–	–	–	–	–	–	–	–	−0.68	−2.87–1.50	1.11	0.540
Age	–	–	–	–	–	–	–	–	0.32	−0.31–0.95	0.32	0.319
*R*^2^ (corr. *R*^2^)	0.34 (0.32)	–	–	–	0.38 (0.36)	–	–	–	0.39 (0.35)			
Rule-breaking with friends												
Low self-control	0.20	0.09–0.31	0.05	<0.001	0.22	0.11–0.33	0.05	<0.001	0.21	0.10–0.32	0.06	<0.001
Guilt	−0.01	−0.13–0.10	0.06	0.826	−0.02	−0.13–0.10	0.06	0.793	−0.01	−0.13–0.10	0.06	0.831
Shame	0.08	−0.01–0.17	0.04	0.057	0.08	−0.01–0.17	0.05	0.082	0.09	−0.01–0.18	0.05	0.066
AEMC	−0.30	−0.54–−0.05	0.12	0.017	−0.27	−0.52–−0.03	0.13	0.029	−0.26	−0.50–−0.01	0.13	0.040
Low self-control × guilt	–	–	–	–	0.00	−0.01–0.02	0.01	0.613	0.00	−0.01–0.02	0.01	0.688
Low self-control × shame	–	–	–	–	0.00	−0.01–0.01	0.01	0.896	0.00	−0.01–0.01	0.01	0.839
Low self-control × AEMC	–	–	–	–	−0.03	−0.06–0.00	0.02	0.053	−0.03	−0.06–0.01	0.02	0.095
Gender	–	–	–	–	–	–	–	–	0.54	−1.15–2.23	0.86	0.533
Age	–	–	–	–	–	–	–	–	0.59	0.11–1.08	0.25	0.017
*R*^2^ (corr. *R*^2^)	0.17 (0.15)	–	–	–	0.19 (0.16)	–	–	–	0.22 (0.18)	–	–	–

Notes. Model 1 including predictor (low self-control) and moderator variables (guilt, shame, AEMC) separately, model 2 including predictor and moderator variables as well as interaction terms; model 3 including predictor and moderator variables as well as interaction terms, and control variables (gender and age), gender is coded as 0 = males, 1 = females, AEMC = Anticipated emotions in moral conflicts; predictor and moderator variables were mean-centered.

**Table 4:** Interaction between unstructured socializing and moral emotions in explaining rule-breaking behavior (own and with friends).

*N* = 169	Model 1				Model 2				Model 3			
	*B*	95% *CI*	*SE*(*B*)	*p*	*B*	95% *CI*	*SE*(*B*)	*p*	*B*	95% *CI*	*SE*(*B*)	*p*
Own rule-breaking												
Unstructured socializing	1.21	0.95–1.47	0.13	<0.001	1.17	0.92–1.41	0.13	<0.001	1.21	0.94–1.48	0.14	<0.001
Guilt	−0.16	−0.29–−0.03	0.07	0.020	−0.25	−0.38–−0.11	0.07	<0.001	−0.25	−0.38–−0.12	0.07	<0.001
Shame	0.26	0.15–0.37	0.05	<0.001	0.32	0.22–0.42	0.05	<0.001	0.33	0.23–0.43	0.05	<0.001
AEMC	−0.51	−0.79–−0.22	0.15	<0.001	−0.42	−0.69–−0.15	0.14	0.003	−0.41	−0.69–−0.13	0.14	0.004
Unstructured social. × guilt	–	–	–	–	−0.07	−0.11–−0.04	0.02	<0.001	−0.07	−0.10–−0.03	0.02	<0.001
Unstructured social. × shame	–	–	–	–	0.07	0.04–0.10	0.02	<0.001	0.07	0.04–0.10	0.02	<0.001
Unstructured social. × AEMC	–	–	–	–	0.04	−0.03–0.10	0.03	0.274	0.04	−0.03–0.11	0.03	0.234
Gender	–	–	–	–	–	–	–	–	−1.06	−2.99–0.87	0.99	0.283
Age	–	–	–	–	–	–	–	–	−0.26	−0.86–0.35	0.31	0.403
*R*^2^ (corr. *R*^2^)	0.46 (0.44)	–	–	–	0.53 (0.51)	–	–	–	0.53 (0.51)	–	–	–
Rule-breaking with friends												
Unstructured socializing	0.70	0.51–0.90	0.10	<0.001	0.68	0.49–0.88	0.10	<0.001	0.63	0.41–0.84	0.11	<0.001
Guilt	−0.04	−0.15–0.06	0.05	0.400	−0.11	−0.21–−0.01	0.05	0.043	−0.11	−0.21–−0.01	0.05	0.041
Shame	0.12	0.04–0.20	0.04	0.003	0.17	0.09–0.25	0.04	<0.001	0.18	0.09–0.26	0.04	<0.001
AEMC	−0.31	−0.53–−0.08	0.11	0.007	−0.24	−0.45–−0.02	0.11	0.030	−0.23	−0.45–−0.01	0.11	0.038
Unstructured social. × guilt	–	–	–	–	−0.05	−0.08–−0.03	0.01	<0.001	−0.06	−0.09–−0.03	0.02	<0.001
Unstructured social. × shame	–	–	–	–	0.05	0.03–0.07	0.01	<0.001	0.05	0.03–0.08	0.01	<0.001
Unstructured social. × AEMC	–	–	–	–	0.05	−0.01–0.10	0.03	0.086	0.05	−0.01–0.10	0.03	0.095
Gender	–	–	–	–	–	–	–	–	0.48	−1.06–2.01	0.78	0.541
Age	–	–	–	–	–	–	–	–	0.32	−0.17–0.80	0.25	0.195
*R*^2^ (corr. *R*^2^)	0.31 (0.29)	–	–	–	0.40 (0.37)	–	–	–	0.40 (0.37)	–	–	–

Notes. Model 1 including predictor (unstructured socializing) and moderator variables (guilt, shame, AEMC) separately, model 2 including predictor and moderator variables as well as interaction terms; model 3 including predictor and moderator variables as well as interaction terms and control variables (gender and age), gender is coded as 0 = males, 1 = females, AEMC = Anticipated emotions in moral conflicts; predictor and moderator variables were mean-centered.

## Data Availability

The data presented in this study are available on request from the corresponding author due to privacy reasons.

## Appendix A

**Table A1:** Descriptive analysis of study variables.

	Original Data					Combined Score Imputed Data				
Variables (No. of Items)	*M*	*SD*	*SE*	Min	Max	*M*	*SD*	*SE*	Min	Max
Guilt (15)	51.9 (*N* = 156)	10.5	0.84	21	75	51.8	10.7	0.83	16	75
Shame (15)	42.1 (*N* = 154)	11.8	0.95	15	75	42.2	11.7	0.90	15	75
AEMC (6)	22.2 (*N* = 163)	4.3	0.34	6	30	22.3	4.3	0.33	6	30
Low self-control (15)	43.4 (*N* = 164)	8.1	0.63	19	62	43.5	8.0	0.62	19	62
Unstructured socializing (4)	10.7 (*N* = 165)	3.8	0.29	4	19	10.7	3.8	0.29	4	19
Own rule-breaking (20)	30.5 (*N* = 158)	8.5	0.68	20	60	30.5	8.4	0.64	20	60
Rule-breaking w. friends (13)	18.2 (*N* = 121)	6.3	0.58	13	39	18.6	5.6	0.43	13	39

Notes: high levels of guilt (1 = unlikely, 5 = very likely) indicate high guilt-proneness; high levels of AEMC (1 = very good, 3 = neither good nor bad, 5 = very bad) indicate feeling bad in case of a selfish decision; high levels of unstructured socializing (1 = never to 5 = always) indicate frequent unstructured leisure activities with friends; high levels of low self-control (1 = not true at all to 5 = very true) indicate low self-control; high levels of own rule-breaking behavior (1 = not true to 3 = accurate or frequently true) indicate more frequent rule-breaking behavior adolescents themselves actively show; high levels of rule-breaking behavior with friends (1 = not true to 3 = accurate or frequently true) indicate frequent rule-breaking committed together with friend.
